# The Mental Health Care Gap among Children and Adolescents: Data from an Epidemiological Survey from Four Brazilian Regions

**DOI:** 10.1371/journal.pone.0088241

**Published:** 2014-02-18

**Authors:** Cristiane S. Paula, Isabel A. S. Bordin, Jair Jesus Mari, Luciane Velasque, Luis A. Rohde, Evandro S. F. Coutinho

**Affiliations:** 1 Programa de Pós-Graduação em Distúrbios do Desenvolvimento, Universidade Presbiteriana Mackenzie, São Paulo, São Paulo, Brasil; 2 Departamento de Psiquiatria, Universidade Federal de São Paulo, São Paulo, São Paulo, Brasil; 3 Departamento de Matemática de Estatística. Universidade Federal do Estado do Rio de Janeiro, Rio de Janeiro, Rio de Janeiro, Brasil; 4 Hospital de Clínicas de Porto Alegre, Universidade Federal do Rio Grande do Sul, Porto Alegre, Rio Grande do Sul, Brasil; 5 Departamento de Epidemiologia e Métodos Quantitativos em Saúde. Escola Nacional de Saúde Pública-FIOCRUZ, Rio de Janeiro, Rio de Janeiro, Brasil; United (Osaka U, Kanazawa U, Hamamatsu U Sch Med, Chiba U and Fukui U) Graduate School of Child Developmen, Japan

## Abstract

**Introduction:**

Worldwide, a minority of disordered children/adolescents receives mental health assistance. In order to improve service access, it is important to investigate factors that influence the process leading to receiving care. Data on frequency and barriers for mental health service use (MHSU) among Brazilian children/adolescents are extremely scarce and are needed to guide public policy.

**Objectives:**

To establish the frequency of MHSU among 6-to-16-year-old with psychiatric disorders from four Brazilian regions; and to identify structural/psychosocial/demographic barriers associated with child/adolescent MHSU.

**Methods:**

Multicenter cross-sectional-study involving four towns from four out of five Brazilian regions. In each town, a representative sample of elementary public school students was randomly selected (sample: 1,721). Child/adolescent MHSU was defined as being seen by a psychologist/psychiatrist/neurologist in the previous 12 months. Standardized instruments measured: (1) children/adolescent characteristics [(1.1) Schedule for Affective Disorders and Schizophrenia for School-Age Children (K-SADS-PL)-psychiatric disorders; (1.2) Ten Questions Screen-neurodevelopment problems; (1.3) two subtests of WISC-III-estimated IQ; (1.4) Academic Performance Test-school performance)], (2) factors related to mothers/main caregivers (Self-Reporting Questionnaire-anxiety/depression), (3) family (Brazilian Research-Companies-Association's Questionnaire-SES).

**Results:**

Only 19.8% of children/adolescents with psychiatric disorder have used mental health services in the previous 12 months. Multiple logistic regression modeling identified five factors associated with lower rates of MHSU (female gender, adequate school performance, mother/main caregiver living with a partner, lower SES, residing in deprived Brazilian regions) regardless of the presence of any psychiatric disorders/neurodevelopmental problems.

**Conclusions:**

Only a small proportion of children/adolescents with psychiatric disorders had been seen by a mental health specialist in the previous 12 months. Structural/psychosocial/demographic factors were associated with uneven access to service for certain groups of children/adolescents. These results call attention to the urgent need to implement programs to help reduce this large unmet mental health need; inequalities must be considered by policy makers when planning strategies to address barriers for care.

## Introduction

The shortages of child mental health services is a priority in the world mental health agenda, [1]–[4]. The consequences of lack of treatment involve societal costs over a lifetime due to negative outcomes that are preventable by early intervention, as well as personal consequences, such as having a worse quality of life and lack of opportunities to achieve full developmental potential [2], [5], [6].

Globally, government spending on child mental health care is insufficient, being disproportionately smaller than funds available for mental health care for older age groups and expenditure on physical health for all age groups [7]–[9]. This situation is particularly relevant in low-middle-income countries (LMIC), where there is a high proportion of young people in the population [2].

Brazil's unified national health system (acronym SUS) has dramatically changed since the 1980's. It has become more regionalized, intersectoral and free for all citizens, but still needs to become more equalized regionally to provide a better quality of service [10]. Moreover, it is necessary to improve human resources and overcome the lack of mental health specialists particularly in smaller towns and in the poorest regions of the country [7], [11], [12]. At the same time, there is an absence of empirical data on the magnitude of mental health service use among Brazilian children and adolescents. This is a pioneering study, being the first multicenter study to be conducted in Brazil on child mental health service use

The main objectives of this study can be summarized as follows: (1) to establish the frequency of mental health service use among children and adolescents with psychiatric disorders from four Brazilian geographical areas; and (2) to identify structural, psychosocial and demographic barriers, other than psychiatric disorders and neurodevelopmental problems, associated with child/adolescent mental health service use in the whole sample.

## Materials and Methods

### Ethics Statement and Procedures

Firstly, local Educational and Health Governmental Agencies were contacted and agreed to support this research in the four selected sites.

Prior to data collection, a team of mental health experts trained and supervised the selected psychologists to conduct the assessments.

Participants were individually assessed in private rooms at buildings rented for this particular survey. Psychologists obtained written informed consent from mothers/primary caregivers before administering the instruments. On average, interviews lasted one and a half hours with mothers/primary caregivers, and 25 minutes with children/adolescents. Data collection took 15 months to be fully completed by December 2011.

Children/adolescents identified as having severe mental health problems were referred to public health services. Local Health Coordinators were committed to assist all cases referred by the study team. The Research Ethics Committee of University of São Paulo approved this study under process number 0301/09.

### Study design

It is a cross-sectional study.

### Setting

Brazil has 195 million inhabitants, and is the fifth largest country in the world [13]. Despite Brazil's impressive recent economic growth, it is still a country that faces serious social inequalities. Its Human Developmental Index (HDI) was 0.76 in 2011 (study data collection year), similar to the Latin America/Caribbean region and slightly higher than the world average (0.69) [14]. HDI is annually published for 186 countries by the United Nations Development Program and represents a push for a broader definition of well-being (http://hdr.undp.org/en/statistics/). HDI is based on three indicators: (i) education (literacy rates and school enrollment rates), (ii) income (GDP per capita), and (iii) life expectancy.

Brazil is composed of five heterogeneous regions, the Southeast and South geographical areas being the two most developed and wealthy. This is a multicenter study, including four towns from four out of five Brazilian geographical areas. The South region was not included because most Brazilian epidemiologic studies on this topic have been carried out in this region. The selection criteria of the four sites were: (1) being near to a State Capital, (2) having a HDI similar to the Brazilian average; and (3) having approximately 30,000 inhabitants, since 84.7% of Brazilian municipalities have less than 50,000 inhabitants [15].

### Sample

Since 2001, it has been mandatory for all Brazilian children/adolescents from 6 years of age to be registered and to complete elementary school (9 years of schooling) [16]. The vast majority of Brazilian students attend public schools, as was the case in the four participant towns: 90% in Caeté (Southeast); 96% in Goianira (Center); 93% in Itaitinga (Northeast); and 95% in Rio Preto da Eva (North) [17].

Local databases of registered students allow for the precise identification of all elementary public school students of any Brazilian municipality. In each site, the local Educational Governmental Agency provided a single list of all public school students from grades two to six. In each town, the study research team randomly selected 500 public school students from the provided list of two to six graders. No school refused to participate.

All selected families received one to five home visits by our research team to arrange an interview to be conducted in private rooms at buildings rented for this particular survey. The reasons for non-participant mothers/main caretakers included: refusal to participate, being absent at least three times at arranged interviews and nobody in (despite a number of visits). No refusal occurred among children from participant mothers/main caretakers. Overall 14% of the 2,000 selected families did not participate in the study and sample loss was not replaced by additional recruitment of participants.

### Variables and Instruments

Variables were measured using seven standardized instruments applied face-to-face to primary caregivers by trained psychologists. In addition, a different psychologist had assessed the children's and adolescents' Intelligent Quotient (IQ) and school performance.

1. The study outcome was defined as mental health service use which meant children/adolescents being seen by a psychologist and/or psychiatrist and/or neurologist for emotional/behavior problems in the previous 12 months. Usually the definition of mental health care includes psychiatrists and psychologists as provider. Nevertheless, it is known that psychiatrists are scarce in many Brazilian small towns, therefore neurologists also deliver medical child mental health care (e.g. treatment of behavior problems and ADHD) and were included as providers in our study. Mental health service use was measured based on a questionnaire developed by the study research team.
2. *Sociodemographic factors* were also collected using a structured questionnaire developed by the study research team that included questions about the child/adolescent's gender, age and area of residence (whether it was urban or rural and in the North/Northeast/Center/South Brazilian region) and the mother/primary caregiver's age, educational level and marital status.
3. *Familial socioeconomic status* was assessed by a questionnaire developed by the Brazilian Association of Research Companies according to family purchasing power [18]. The instrument is one of the most used in Brazil and takes into account, among other factors, the number of home appliances and the education level of the head of the household. Total scores determine the socioeconomic status of families classifying them in five social classes (A, B, C, D, E). In the current study, classes were grouped in three categories: Middle-High (A, B), Middle-Low (C, D), and Low (E).
4. *Psychiatric disorders in children and adolescents* were evaluated using the Schedule for Affective Disorders and Schizophrenia for School-Age Children/Present and Lifetime Version (K-SADS-PL) based on mother/main caregiver report. K-SADS-PL is a semi-structured psychiatric interview that ascertains diagnostic status based on DSM-IV criteria including five diagnostic groups: (i) affective disorders (depression disorders [major depression, dysthymia] and mania, hypomania); (ii) psychotic disorders; (iii) anxiety disorders (social phobia/agoraphobia/specific phobia/obsessive-compulsive disorder/separation anxiety disorder/generalized anxiety disorder/panic disorder/posttraumatic stress disorder); (iv) disruptive behavioral disorders (ADHD/conduct disorder/oppositional defiant disorder); and (v) substance abuse, tic disorders, eating disorders, and elimination disorders (enuresis/encopresis) [19]. Evidence of convergent validity of the Brazilian version of the K-SADS-PL was found by comparison to the Child Behavior Checklist (CBCL) [20]. In addition, K-SADS-PL is one of the few validated diagnostic instruments in Brazil to measure psychiatric disorders in children and adolescents. For the present study, any psychiatric disorder was defined as children/adolescents having one or more of the disorders included in these five psychiatric diagnostic groups (excluding enuresis/encopresis) in the past 12 months.
5. The Ten Question screen (TQ) consists of ten yes-or-no questions that cover functional limitations in the domains of speech, cognition, hearing, vision, motor and physical impairment and also addresses seizures. This instrument, applicable for children aged 2 to 9 years of age based on parent report, was validated in several countries and has been one of the mostly widely used screening tool for childhood disability in LMICs [21], [22]. Although the TQ has been developed for children from 2 to 9 years of age, it assesses symptoms of chronic conditions related to abnormal neurodevelopment; therefore, it was used to evaluate all children/adolescents in the current study.
6. The IQ of children/adolescents was evaluated according to the Wechsler Intelligence Scale for Children (WISC-III). The WISC-III is an individually administered intelligence test for 6–16 year-old children/adolescents with good psychometrics proprieties and has been validated in Brazil [23]–[25]. WISC-III is considered one of the best instruments to assess cognition in children/adolescents, but it is too long to be used in epidemiological investigations. At the same time, previous studies have shown a high correlation between reduced versions (with 2, 4 or 6 subtests) and the full version of the instrument [26]. Thus, in the current study two WISC-III's subtests were used (Vocabulary and Blocks) to establish children/adolescents' estimated IQ [27].
*Neurodevelopmental problems* were defined as a combination of two items from the TQ (“Does the child have weakness and/or stiffness in the limbs and/or difficulty in walking or moving his arms?” and “Does the child sometimes have fits, become rigid, or lose consciousness?”) and/or children/adolescents' IQ lower than 70 (considered intellectual disability) [28]
7. The Self-Report Questionnaire (SRQ-20) was applied to evaluate *mother/main caregiver's anxiety and/or depression*. SRQ-20 is a screening instrument developed by the WHO with 20 yes-no items to identify symptoms that may be indicative of common mental disorders, and is especially recommended for low/middle-low income countries [29]. The Brazilian version of SRQ-20 has good validity and high reliability with a cut-off point of 7/8 [30].
8. School performance was assessed by the Academic Performance Test (*Teste de Desempenho Escolar*- TDE), a psychometric instrument addressing fundamental skills for school performance, specifically writing, arithmetic and reading. It was developed and validated for the Brazilian population indicating the areas of school performance that are preserved or impaired in children [31]. TDE classifies children/adolescents in five categories that in the current study were grouped in three: Average/Superior; Middle-Low and Low.

### Statistical Analysis

For descriptive purposes, we firstly calculated the distribution of structural, psychosocial and demographic variables stratified by the four cities. Additionally, we estimated the frequency of mental health service use for those with and without psychiatric disorders.

In order to investigate the variables associated with the use of mental health services (outcome variable), a sequence of bivariate analyses were performed including all structural, psychosocial and demographic variables, one by one, with mental health service use. Those variables presenting p-values<than 0.20 in this stage of the analysis were then selected for inclusion in an initial multiple logistic regression model [32]. As the objective was to identify factors associated with mental health service use other than psychiatric disorders and neurodevelopmental problems, the models were adjusted for these two variables. The final model retained the variables with p-values≤0.05, but those between 0.06 and 0.10 were interpreted as having borderline statistical significance. Linear trend was also tested for ordinal variables.

To evaluate the impact of missing values of the psychiatric disorder variable on our estimates, we carried out a sensitivity analysis. Initially, missing cases were grouped in a specific category to investigate the existence of a pattern in its association with the outcome variables that could suggest the presence of bias (not missing at random). Moreover, two opposite scenarios were tested: (i) all missing cases being positive for psychiatric disorder and (ii) all missing cases being negative. The parameters of the two models under these hypothetical scenarios were compared to the final model to check if the exclusion of this set of missing individuals would change the conclusions of the study. Linear trend was also tested for ordinal variables using chi-square tests.

Validation of the model was investigated by plotting and checking (i) the residuals against the fitted values, (ii) Normal Q-Q plot, (iii) plot of Cook's distances, and (iv) plot of residuals against leverages [33]. The Hosmer-Lemershow test was used to assess goodness-of-fit [34]. Multicollinearity was verified calculating the Variance Inflation Factor (VIF), considering the cutoff >10 as an indicator of collinearity [35]. All analyses were conducted using SPSS 20 and R softwares (version 3.0.1).

## Results

This study comprised random samples from four towns from four different Brazilian regions. From the 2,000 selected families, mother/main caretakers and students response rates differed by region: 90.4% (Southeast), 89.8% (Central), 81.8% (North), and 81.2% (Northeast). In relation to all participant children/adolescents (6–16 years) mothers were the informants for 90.1% of them whilst grandmothers, fathers and aunts provided answers for 9.9% of the total sample.

Considering the entire sample, the prevalence of any psychiatric disorder was 13.1% and of neurodevelopmental problems was 10.4%. At the same time, the mental health services use in the whole sample (independently of having or not psychiatric disorder/neurodevelopmental problems) was 9.2%.

Table 1 describes samples by town of residence and it is noticeable that they were different in almost all characteristics except children/adolescents gender. As these differences were identified, town of residence was included as one of the potential factors related to MHSU.

**Table 1:** Structural, psychosocial and demographic characteristics of the sample by site (N = 1,721).

Sample characteristics by domain	Town from Southeast (N = 458)	Town from Center (N = 449)	Town from Northeast (N = 406)	Town from North (N = 408)	p
Child/adolescent					
Gender					
Male (%)	53.3	52.6	50.7	55.3	0.63⊝
Age (years)					
Mean (SD)	8.8 (1.58)	9.1 (1.80)	10.0 (1.99)	10.3 (1.95)	<0.01£
Place of residence					
Urban (%)	89.7	100.0	45.3	70.4	<0.01⊝
School performance (%)*					
Average or superior (reference)	29.2	39.2	32.5	13.2	<0.01⊝
Middle-Low	17.5	22.3	18.7	17.6	
Borderline or low	53.3	38.5	48.8	68.2	
Mental health service use (past 12 months)					
Yes (%)	16.4	6.7	7.6	5.6	<0.01⊝
Mother/primary caregiver					
Age**					
Mean (SD)	36.0 (7.91)	33.8 (7.30)	36.8 (9.00)	36.49 (9.84)	<0.01£
Education (years of schooling)#					
Mean (SD)	8.0 (4.01)	7.0 (3.45)	4.9 (3.70)	6.7 (3.89)	<0.01£
Marital status					
Married/living with a partner (%)	75.1	78.2	78.8	83.4	0.03⊝
Anxiety/depression (SRQ-20 total score)§					
Yes (>7)	29.7	42.3	35.6	33.2	<0.01⊝
Family					
Socioeconomic status (%)					
Low	1.7	2.0	8.6	11.0	<0.01⊝
Middle-low	80.6	91.5	89.2	82.4	
Middle-high (reference)	17.7	6.5	2.2	6.6	

*2 missing;

**6 missing;

# 1 missing;

§ 3 missing.

⊝ chi-square test;

£ ANOVA test.

Unsurprisingly, there is an association between children/adolescents' any psychiatric disorders and mental health service use. However, it is important to stress that among those presenting any psychiatric disorder only 19.8% had used mental health services in the previous 12 months (table 2). The vast majority of mental health consultations were offered by psychologists (84.9%), in comparison to 20.9% from psychiatrists and 18.8% by neurologists (data not presented).

**Table 2:** Use of mental health service according to psychiatric disorders in the whole sample (N = 1,721).

K-SADS diagnoses	Mental Health Service Use (past 12 months)		p
	Yes	No	
	N (%)	N (%)	
Any Psychiatric Disorder*			
No (reference)	107 (7.6)	1303 (92.4)	<0.01
Yes	42 (19.8)	170 (80.2)	
Main diagnostic groups**			
None (reference)	107 (7.6)	1303 (92.4)	
Only Affective Disorders	1 (20.0)	4 (80.0)	0.33
Only Anxiety Disorders	15 (17.2)	72 (82.8)	<0.01
Only Disruptive Disorders	13 (20.3)	51 (79.7)	<0.01
Only Eating/Tic/Substante Use Disorders	1 (9.1)	10 (90.9)	0.58
Only Psychotic Disorders	0 (0.0)	1 (100.0)	NA
Comorbidity&	9 (30.0)	21 (70.0)	<0.01

*99 missing due to shortage of information provided by mothers/caregivers;

** 113 missing due to shortage of information provided by mothers/caregivers;

NA not applicable;

& two or more positive diagnostic groups.

In relation to the five main psychiatric diagnostic groups, it is noticeable that mental health service use was associated with pure anxiety disorders, pure disruptive disorders and the presence of two or more diagnostic groups (comorbid group). In addition, chi-square tests (p-values not presented) showed no statistically significant difference in mental health service use rates among diagnostic groups considered alone or in combination.

Besides the association among psychiatric disorders and neurodevelopmental problems with mental health service use, we investigated 9 potential associations with three different sets of variables (child/adolescent; mother/primary caregiver; family). According to the bivariate analyses (table 3), regardless of any psychiatric disorders/neurodevelopmental problems, eight out of 9 structural/psychosocial/demographic variables were associated with lower mental health service use: (a) related to child/adolescent (being a younger child/adolescent, being female, living in Northeast/Central/Northern regions and having better school performance); (b) related to mother/primary caregiver (having a lower level of education, living with a partner and having less anxiety and/or depression symptoms); and (c) related to family (being from a lower socioeconomic group).

**Table 3:** Bivariate analyses showing children/adolescent, mother/primary caregivers and family potential associated factors with mental health service use (N = 1,721).

	Mental Health Service Use (past 12 months)			
	Yes N (%)	No N (%)	OR(IC 95%)	p
Child/Adolescent				
Age (years)				
Mean (SD)	9.70 (1.93)	9.48 (1.93)	NA	0.14
Gender				
Female	58 (7.2)	751 (92.8)	0.62 (0.44;0.87)	<0.01
Male (reference)	101 (11.1)	811 (88.9)		
Town of residence				
North	23 (5.6)	385 (94.4)	0.31 (0.19;0.50)	<0.01
Center	30 (6.7)	419 (93.3)	0.37 (0.23;0.57)	<0.01
Northeast	31 (7.6)	375 (92.4)	0.42 (0.27;0.66)	<0.01
Southeast (reference)	75 (16.4)	383 (83.6)	1	
School Performance*				
Average or Superior	39 (6.4)	566 (93.6)	0.62 (0.41;0.94)	0.02
Median-Low	28 (8.4)	304 (91.6)	0.83 (0.51;1.33)	0.42
Borderline or Low (reference)	78 (10.0)	704 (90.0)	1	
Psychiatric Disorders**				
No	107 (7.6)	1303 (92.4)	0.33 (0.23;0.49)	<0.01
Yes (reference)	42 (19.8)	170 (80.2)		
Neurodevelopmental Problems#				
No	120 (7.9)	1407 (92.1)	0.31 (0.21;0.47)	<0.01
Yes (reference)	38 (21.3)	140 (78.7)		
Mother/Primary caregiver				
Age (years)§				
Mean (SD)	36.5 (9.19)	35.6 (8.54)	NA	0.25
Education¶				
Mean (SD)	7.1 (4.14)	6.7 (3.93)	NA	0.15
Marital Status				
Living with a partner	111 (8.2)	1245 (91.8)	0.58 (0.41;0.86)	<0.01
Single, Divorced or Widow (reference)	48 (13.2)	317 (86.8)		
Anxiety/depression (SRQ-20 total score)&				
No (≤7)	93 (8.4)	1020 (91.6)	0.74 (0.53;1.06)	0.08
Yes (>7) (reference)	66 (10.9)	539 (89.1)		
Family				
Socioeconomic status				
Low	4 (4.1)	93 (95.9)	0.30 (0.07;0.97)	0.03
Middle-low	137 (9.3)	1342 (90.7)	0.72 (0.42;1.29)	0.22
Middle-high (reference)	18 (12.4)	127 (87.6)	1	

*2missing;

**99 missing;

# 16 missing;

§ 6 missing;

¶ 1 missing;

& 3missing;

NA = Not applicable.

In order to further investigate the role of these variables independently of the presence of psychiatric disorders and neurodevelopmental problems, multiple logistic regression models were fitted. As mentioned in the methods section, variables with p-values lower than 0.20 in the bivariate analysis were entered in the initial model. All entered variables except children/adolescents' age, and educational level of the mother/primary caregiver were retained in the final model. Children/adolescents of female gender from the less developed regions (Northeast, Central or Northern) with better school performance (average/superior or median-low), from families where mother/primary caretaker live with a partner and had lower socioeconomic status (middle-low or low), were associated with lower rates of mental health service use, independently of their status concerning psychiatric disorders and neurodevelopmental problems (table 4). Furthermore, in the two variables with three categories (school performance and socioeconomic status), we observed a linear trend for service use (p<0.01).

**Table 4:** Final model of multiple logistic regression identifying children/adolescent, mother/primary caregivers and family associated factors with mental health service use (N = 1,721).

Factors	OR (CI 95%)	p
Final Model		
Child/adolescent		
Female gender	0.65 (0.46–0.92)	0.02
Town of residence		
North	0.22 (0.13–0.37)	<0.01
Center	0.59 (0.33–1.04)	0.07
Northeast	0.66 (0.37–1.20)	0.17
Southeast (reference)	1	
School performance		
Average/superior	0.52 (0.34–0.79)	<0.01
Median-low	0.76 (0.45–1.29)	0.31
Borderline or low (reference)	1	
Mother/primary caregiver		
Living with a partner	0.60 (0.41–0.88)	0.01
Family		
Socioeconomic status		
Low	0.30 (0.91–0.98)	0.05
Middle-low	0.39 (0.14–1.13)	0.08
Middle-high class (reference)	1	

The sensitivity analysis for the effect of missing values found no suggestion of bias due to the lack of information in 99 children/adolescents. Thus, we compared the estimates and confidence intervals of 3 different models: 1) model A, when individuals with missing values in the variable any psychiatry disorder were excluded; 2) model B, when all participants with missing values were coded 1 (psychiatric disorders/neurodevelopmental problems = yes); and 3) model C, when the missing values were coded 0 (psychiatric disorders/neurodevelopmental problems = no). In this analysis (data not shown), the estimates of all the variables retained in the final model A, did not differ significantly (overlap CI 95%) from the ones estimated in models B and C. Finally, tests of goodness-of-fit (p = 0.79) and collinearity did not suggest any inadequacy in the final model.

## Discussion

This is the first study conducted in four Brazilian regions addressing barriers on child/adolescent MHSU. This is especially important in a large country like Brazil where socio-economic and demographic differences vary greatly between the North and South of the country as was demonstrated in table 1.

The current study showed that only 19.8% of children/adolescents with any psychiatric disorder had been seen by a mental health specialist in the previous 12 months. This rate was similar to the rate obtained among children/adolescents with disorders from specific diagnostic groups (e.g. only anxiety, only disruptive disorders). The low frequency of specific diagnostic groups (e.g. only affective, only eating/tic/substance use and only psychotic disorders) meant it was not possible to produce any statistically significant data for association with MHSU.

Considering the total number of children/adolescents that used mental health services in the past 12 months (N = 149), the vast majority of appointments (84.9%) were offered by psychologists, reflecting the Brazilian reality where: (i) there are three times more psychologists than psychiatrists or neurologists, (ii) the total number of child and adolescent psychiatrists is still small (about 300 in 2007) and concentrated in big cities; and (iii) there is one psychiatrist per 75 primary care units versus one psychologist per 7 units [11], [12], [36], [37].

Many studies about child/adolescent mental health service use in high-income countries have been carried out since early 1990s. Although the figures were not consistent in their estimation of the scale of the problem, all of them have highlighted the unmet need for mental health services. In 2013, a study conducted with 1,445 students around the US estimated that almost half (45.3%) of those with DSM-IV disorders received mental health treatment in the preceding year [38]. Another notable survey published in 2010 with a nationally representative sample of the US population (3,024 children; 8–15 year-olds) showed that approximately half (50.6%) of those with any psychiatric disorder had received treatment from a mental health professional in the past-year [3]. In Finland, one of the most important nationwide research studies about mental health service use is the “From a Boy to a Man study”. In 2004, authors published data on the 10-years follow-up stage of the study with 2,316 18 year-old boys. Results showed that only 4.5% of those with mild and 15.7% with moderate/severe behavioral, emotional or relational problems had used services in the previous 12 months [39].

On the other hand, data on child/adolescent mental health service use in LMIC, including Latin America, are scarce and there is still a need for more study in this area. As far as we know, figures from Puerto Rico and Mexico are the only ones available for this region. In Puerto Rico, there are several epidemiological studies on the child mental health field, including reliable data on service use. An often cited study published in 2004, encompassing a representative sample of Puerto Ricans aged 4–17 (N = 1,886) estimated that mental health treatment was received by 25.7% of cases with any DSM-IV psychiatric disorder and no global impairment in the past year, 39.5% of those with any psychiatric disorder and less severe global impairment, and 49.6% children/adolescents with any psychiatric disorder and more severe global impairment [40].

In Mexico, several studies on mental health services for adults had been published [41], [42]. In 2008, a Mexican study presented data from a probabilistic sample of 3,005 adolescents (12 to 17 year-olds) from the metropolitan area of Mexico City. The authors verified that 13.7% of those with psychiatric disorders had received some type of mental health treatment in the preceding year [43].

Data produced around the world indicates that most children/adolescents with mental health problems do not have access to treatment and several barriers involved in this have been highlighted in the literature, inadequate funding is one of the most important obstacles for the development of services, especially for young people; as well as lack of government commitment and overcentralisation [2], [44], [45]. In Brazil, financial resources represent one of the most important barriers since only 2% of the total Brazilian health budget (in comparison to 6–12% in high-income countries) is allocated to mental health, which is translated in US$ 3.00 per capita per year [45].

In addition, structural, psychosocial and demographic factors [1], [40], [46], [47], stigmatization, lack of trained workforce and lack of awareness among parents/teachers/health professionals also play an important role [7], [44], [48]. In the current study we identified factors from three domains that decreased the likelihood of receiving mental health assistance, regardless of having any psychiatric disorders or neurodevelopmental problems: (i) children/adolescents of female gender, with average/superior or median-low school performance and living in the less developed regions of the country; (ii) mother/primary caregiver's lower educational level and living with a partner; and (iii) families of lower socioeconomic status.

Female gender has been described in most studies as a factor associated to lower rates of mental health assistance [3], [46], [49], as it was identified in the present study. Several studies show that girls go less frequently to services and they get diagnosis later than boys [50]. Many authors argue that boys, during childhood/early adolescence present more externalizing symptoms, which are easier to recognize and consequently to seek help for [47]. In our sample almost two-thirds of the participants were 6–10 year-olds, which make this hypothesis plausible. However, some recent studies found that girls with the same type of diagnosis and severity of symptoms were less referred to services than boys [50]. Another study pointed out that girls with ADHD, unlike boys, were referred to services only when they have comorbidity with disruptive behavior [51]. Both studies indicated that girls must be more impaired than boys to be referred for assistance. The current study, in agreement with many others, calls attention to the fact that girls have unequal access to mental health care.

This study also found that living in less developed regions of Brazil (The Northeast, Center or North) decreased the likelihood of receiving mental health consultations. This finding is not unexpected because the starting point for service provision depends on health unit availability and the service system organization [47], [52]. The Brazilian healthcare system (SUS) aims to provide universal access to health services for all Brazilian citizens and the mental health system is entirely integrated with SUS. Despite SUS being readily available for all, financial and human resources are extremely unequally distributed among regions [10], [53]. Likewise, there is a growing and significant portion of the Brazilian population who use private health insurance (26% in 2008) [10], [54], largely from higher social classes. This uneven healthcare system generates obstacles comparable with those observed in countries with an insurance-based model, such as United States and Australia [47]. Overall, Minas Gerais State, where the town representing the Southeast region is located, has some better indicators than the other three less developed States involved in the study. For example, it has 12 Psychosocial Community Care Centers for Children and Adolescents (acronym CAPSI) versus six in Ceará State, two in Goiás, and none in Amazonas [55]. It is important to highlight that CAPSI is “the main source of healthcare for individuals younger than 24 years of age with severe and persistent mental illness and/or with a high level of impairment” in Brazil [7], [56].

In our study, better school performance was also associated with lower probability of receiving mental health assistance in the past 12 months. This association has been identified in many cross-sectional [40], [47] and follow-up studies [39], [57]. This indicates that teachers might be key elements in the detection and referral process [58], since school problems do not seem to raise parent's recognition psychiatric disorders among their kids [47]. Thus, the present study, as previous ones, showed that children/adolescents with good school performance have a greater chance of not being detected and consequently do not receive mental health care.

Additionally, children/adolescents from families where mother/primary caretaker live with a partner had a lower chance of being seen by a psychologist, psychiatrist or neurologist for emotional and/or behavior problems. This result indicates that single, divorced or widowed mothers/primary caregivers sought help more often than the ones living with a partner. Several studies have shown that the burden related to main caregivers' experience in taking care of their child plays an important role in recognition of a problem and the help seeking process [47], including single parenthood [46], [59].

In our sample, children/adolescents of families from the middle-low and low classes received less mental health assistance than children/adolescents from the middle-high class. Overall, it is known that poor families have less access to services [60], but the pathway is not that simple because it is extremely dependent on type of insurance and country's healthcare system. It seems that when financial barriers are removed by providing ready access to care for all, socioeconomic factors may not play a significant role in help-seeking behavior or mental health service use [46], [47], [52]. As mentioned before, the Brazilian healthcare system is free for all but very unequal, particularly in relation to specialized care [53], [54]. Specialized professionals are less available in the poorest neighborhoods and those living in these areas are also less likely to have access to private health insurance. Hence, the inequality of health access reflects the Brazilian social inequalities.

The current study has some strengths including: (i) giving the first estimates of the magnitude of the gap in child mental health treatment in four Brazilian regions; (ii) psychiatric disorders were assessed by K-SADS-PL, a standardized diagnostic criteria that facilitate comparisons with international studies and one of the most recognized instruments for this age group; (iii) the study was able to cover a broad spectrum of factors, including structural, psychosocial and demographic ones. Nevertheless, some limitations must be addressed: (i) all information related to service use was provided by mothers/primary caregivers and it was based on a questionnaire developed by the study research team instead of some service use instrument; (ii) not all factors potentially associated with MHSU such as stigmatization and lack of awareness among parents and teachers were explored in the current study; (iii) information on service use was limited, not providing details about the pathway of mental health assistance, such as number of consultations; (iv) K-SADS-PL was applied to mothers/main caregivers without interviewing adolescents about their symptomatology.

## Conclusions

Only a small proportion of children/adolescents with any psychiatric disorder had been seen by a mental health specialist in the previous 12 months. This result is vital to call policy makers' attention to the urgent need to implement programs to help reduce this large unmet mental health need. In addition, five structural, psychosocial and demographic factors were identified as barriers to service access, regardless of psychiatric disorders and/or neurodevelopmental problems in the child/adolescent: being of female gender, being from deprived Brazilian regions, having adequate school performance, being the child of a mother/primary caregiver who is living with a partner and lower socioeconomic status. This data reveals unequal access to services for certain groups of children/adolescents and requires specific governmental actions, such as: (i) public campaigns to increase awareness among parents and teachers to avoid the unbalanced access to mental health care between girls and boys; (ii) more financial investment in infrastructure and human resources for the health sector in order to equally benefit all Brazilian regions and reduce inequalities among socioeconomic groups; and (iii) training programs for teachers to increase their capacity to recognize children in need of mental health assistance regardless of their school performance.
